# Systemic Redox Imbalance in Chronic Kidney Disease: A Systematic Review

**DOI:** 10.1155/2016/8598253

**Published:** 2016-08-03

**Authors:** Konstantina P. Poulianiti, Antonia Kaltsatou, Georgia I. Mitrou, Athanasios Z. Jamurtas, Yiannis Koutedakis, Maria Maridaki, Ioannis Stefanidis, Giorgos K. Sakkas, Christina Karatzaferi

**Affiliations:** ^1^Department of Physical Education & Sport Science, University of Thessaly, Karyes, 421 00 Trikala, Greece; ^2^Department of Kinesiology, Institute for Research and Technology-CERTH, Thessaly, Karyes, 421 00 Trikala, Greece; ^3^School of Sports, Performing Arts & Leisure, University of Wolverhampton, Wolverhampton WV1 1LY, UK; ^4^Department of Physical Education & Sport Science, National and Kapodistrian University of Athens, 172 37 Athens, Greece; ^5^Department of Nephrology, School of Medicine, University of Thessaly, 411 10 Larissa, Greece; ^6^Faculty of Sport and Health Sciences, University of St Mark and St John, Plymouth PL6 8BH, UK

## Abstract

Patients with chronic kidney disease (CKD) experience imbalance between oxygen reactive species (ROS) production and antioxidant defenses leading to cell and tissue damage. However, it remains unclear at which stage of renal insufficiency the redox imbalance becomes more profound. The aim of this systematic review was to provide an update on recent advances in our understanding of how the redox status changes in the progression of renal disease from predialysis stages 1 to 4 to end stage 5 and whether the various treatments and dialysis modalities influence the redox balance. A systematic review was conducted searching PubMed and Scopus by using the Cochrane and PRISMA guidelines. In total, thirty-nine studies met the inclusion criteria and were reviewed. Even from an early stage, imbalance in redox status is evident and as the kidney function worsens it becomes more profound. Hemodialysis therapy* per se* seems to negatively influence the redox status by the elevation of lipid peroxidation markers, protein carbonylation, and impairing erythrocyte antioxidant defense. However, other dialysis modalities do not so far appear to confer advantages. Supplementation with antioxidants might assist and should be considered as an early intervention to halt premature atherogenesis development at an early stage of CKD.

## 1. Introduction

In the literature there is strong evidence suggesting that uremia, which develops in tandem with renal deterioration, is accompanied by an enhanced state of oxidative stress, which in turn is associated with higher risk of developing cardiovascular disease (CVD) and mortality [1]. The presence of oxidative stress in uremic patients is manifested by increases in the levels of several oxidative damage markers and by a parallel reduction in their antioxidant defense capacity [2, 3]. In chronic kidney disease (CKD), this state of redox imbalance is linked to additional pathological complications that CKD patients present with, such as malnutrition, anemia, and cardiovascular disease including atherosclerosis [2, 4, 5].

CVD remains the leading cause of death in CKD patients, with oxidative stress being implicated in the onset and the development of atherosclerosis, via mechanisms such as oxidation of low density lipoprotein (LDL) [6]. It is reported that levels of lipid and protein peroxidation markers, such as malondialdehyde (MDA), advanced glycosylation end product (AGE), and advanced oxidation protein products (AOPPs), are elevated in CKD patients, with some studies indicating a correlation between some lipid peroxidation markers and intima-media thickness [7–9]. Lipid peroxidation could be described as a process where oxidants, such as free radicals, assault lipids especially in membranes [10]. Additionally, regarding erythrocyte membrane integrity, modifications due to oxidation and the increased membrane rigidity could play a key role in shortening erythrocyte half-life compounding the development of anemia [11], which characterizes these patients. Furthermore, a disturbance of the glutathione antioxidant system, including overall compromised blood levels of reduced glutathione (GSH), increased oxidized glutathione (GSSG), and increased erythrocyte glutathione peroxidase (GPx) and glutathione reductase (Gr reductase) activities, has been reported in CKD [12, 13]. Likewise, a decrease in catalase (CAT), a key enzyme for the detoxification of hydrogen peroxide and organic hydroperoxide, accompanied by increased nitric oxide (NO) inactivation and protein nitration by reactive oxygen species (ROS) [14], has been linked to the pathogenesis of CKD related hypertension via a dysregulation of NO function. Further, at the level of the kidney* per se*, redox disturbances can have direct effects on the afferent arteriole function (e.g., vasoconstriction) and tubuloglomerular feedback (e.g., affecting macula densa cell function or signaling), contributing to the development of hypertension [15].

A variety of mechanisms have been implicated in the generation of oxidative stress in uremic patients, such as antioxidant deficiency and chronic inflammation [16, 17] including advanced glycation mediated disturbances of glomerular homeostasis as disease progresses [18]. In addition, for the end stage renal disease (ESRD) patients, neutrophil activation during hemodialysis (HD) therapy [19] and dialysis treatment* per se* contribute to the increase of oxidative damage observed in patients with CKD [20–22]. Four main factors have been proposed to be responsible for the harmful effects of oxidative stress in patients under HD: the uremic milieu, the HD treatment* per se*, the hemoincompatibility of dialysis system, and the concomitant drug treatment [23].

Until today most studies examining the redox status in CKD patients focused on the end stage of the disease. However, it remains unclear when during disease progression the redox imbalance manifests itself and which one of its components, defense capacity or ROS overproduction, becomes critical first. Thus the aim of this systematic review was to provide an update on recent advances in our understanding of how the redox status changes in the progression of CKD from predialysis stages (1 to 4) to end stage 5 and whether various treatments positively or negatively influence the balance between oxidant and antioxidant mechanisms.

## 2. Methods

The current review includes original studies reporting on oxidative stress markers in CKD patients (including predialysis and ESRD, on hemodialysis or peritoneal dialysis). PubMed and Scopus databases were searched by two reviewers according to the Cochrane and PRISMA guidelines [24, 25] in order to identify publications in English related to oxidative stress in blood of uremic patients. A comprehensive literature search was conducted from September 2015 until November 2015. The time frame the studies were searched was from January 1995 until November 2015. Studies were selected using inclusion and exclusion criteria. We included studies that met the following criteria: they assessed oxidative stress markers in the blood of uremic patients (hemodialysis, peritoneal dialysis, or predialysis); they used blood samples; they addressed randomized control trials, controlled trials, or clinical trials designed to evaluate oxidative stress in blood of uremic patients on HD and PD therapy or not; they were written in English. Exclusion criteria included the following: studies of patients with untreated cardiovascular disease, metabolic syndrome, hyperparathyroidism and hypertension, kidney transplantation, atherosclerosis, and cancer. Moreover, case studies, dissertations, letters, studies published only in abstract form, and studies that were unavailable in English were excluded from the analysis as well. Firstly, titles and abstracts were assessed, followed by assessment of full-text copies of the articles. The initial screening was followed by an extra search in order to identify only the studies that dealt with oxidative stress in uremic patients.

## 3. Overall Results

**Figure 1:**
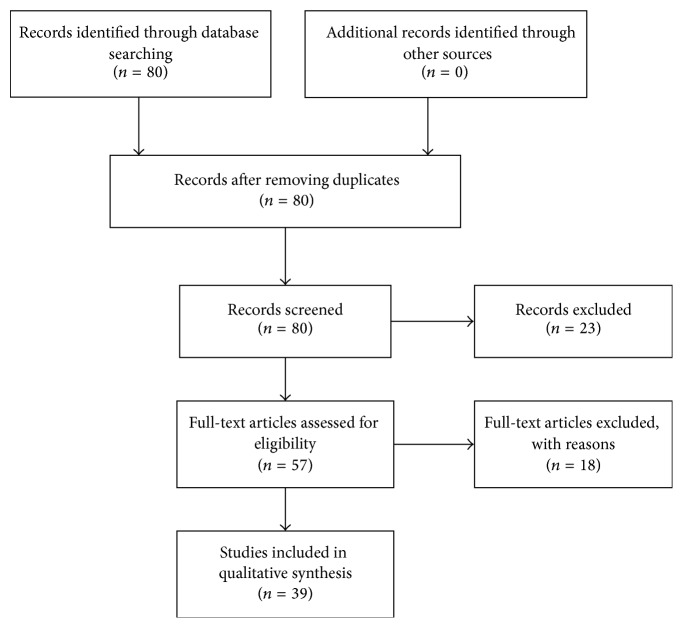
Progress of the literature screening.

The literature search identified a total of 80 articles. Of these, 39 studies met the inclusion criteria, which were set in this review (Figure 1). Articles were excluded for the following reasons: 23 studies correlated CKD with factors which were irrelevant to oxidative stress and thus were out of our interest or dealt mainly with other comorbidities such as diabetes, cardiovascular disease, and hyperparathyroidism. One study examined acute chronic liver disease instead of CKD. Seven studies did not measure oxidative stress biomarkers, to three, despite efforts, we gained no access, one was nonrandomized study, one was not written in English, and 5 did not have detailed methodology.

From the included studies, three evaluated oxidative stress markers in blood of predialysis patients compared to healthy individuals and four studies dealt with several types of interventions in these patients. Ten studies examined oxidative stress in HD patients compared to healthy individuals and eight studies assessed oxidative stress before and after HD session. Seven studies reported some interventions affecting the redox status of HD patients. Two studies examined oxidative stress biomarkers in patients undergoing other types of HD and one study reported an intervention that took place in these patients. One study examined simultaneously oxidative stress in predialysis, HD dialysis, and PD dialysis compared to healthy individuals. One study examined oxidative stress markers in predialysis patients compared to controls, in HD patients compared to controls, and in HD patients before and after an HD session. Two studies evaluated oxidative stress in HD patients compared to controls and in HD patients before and after an HD session. For these reasons data from these last four studies were included in more than one Table.

## 4. Analysis and Discussion

We used the CKD severity staging reported by the authors themselves, or when not available, to facilitate discussion, we adopted the definition of CKD stages based on measured or estimated Glomerular Filtration Rate (GFR), according to KDIGO 2012 guidelines for CKD [26]. According to these guidelines, there are five stages, where kidney function is considered as normal in stage 1 and is progressively reduced as progressing from stages 2 to 5. Each section reporting on the analysis of our systematic review's findings concludes with discursive commentary using relevant information from other literatures.

### 4.1. Predialysis Patients and Redox Status

Only 5 studies examined the redox status in predialysis CKD patients. These studies evaluated the oxidative damage as it was assessed by the lipid peroxidation biomarker MDA and the prooxidant enzyme xanthine oxidase (XO) as well as the antioxidant defense as it was assessed by GSH concentration and superoxide dismutase (SOD) and CAT and GPx activities (Table 1).

Regarding lipid peroxidation damage, four studies measured MDA levels in plasma or red blood cells (RBC) of CKD patients. Specifically, Peuchant et al. 1996 [11] found increased levels of RBC-free MDA in 16 patients with stage 5 CKD compared to age-matched controls. Additionally, Sahni et al. [27] found increased RBC MDA levels in 60 patients with severe CKD (near stage 5) and in 85 patients with moderate CKD compared to age-matched controls, with RBC MDA levels being significantly higher in the severe compared to moderate disease group. MDA levels were also evaluated in plasma and Papavasiliou et al. [28] found them increased in 23 patients with stages 3–5 CKD, compared to age-matched controls. In the same study 13 patients on stages 1-2 CKD exhibited a tendency for higher MDA levels compared to healthy controls but values' differences did not reach statistical significance. In contrast, patients with stages 1-2 exhibited significantly lower MDA levels compared to the patients with stages 3–5 CKD [28]. Bober et al. 2010 [29], measuring TBARS (which are expressed as equivalents of MDA levels), reported higher plasma levels in 21 predialysis CKD patients on conservative treatment and age-matched healthy controls. However, it was not clear if results were statistically significant nor was there information on the predialysis stage of those patients. Choi et al. [30] reported that xanthine oxidase (XO) activity was increased in the plasma of 16 patients with stage 4 CKD (almost ~1.5-fold compared to values of healthy individuals), indicating an increase in free radicals generation.

Regarding antioxidant defense in predialysis patients, four studies evaluated several antioxidant molecules and enzymes. Sahni et al. 2012 [27] found that the increase in lipid peroxidation biomarkers in predialysis patients was accompanied by a reduction in antioxidant enzymes activity being reflected by reduced GSH, SOD, and CAT levels in both severe and moderate predialysis patients, compared to healthy individuals [27]. On the other hand, in the study of Bober et al. [29], higher levels of GSH were found in CDK patients compared to healthy individuals but as it was referred above the stage of patients' disease was not mentioned in the study. However, a study by Peuchant et al. [11] did not observe any changes in the activities of the free radical scavenging enzymes GPx, SOD, and CAT in the erythrocytes of CKD patients compared to healthy individuals. Significantly higher levels of erythrocytes GPx activity have also been observed in both patients with stages 1-2 and stages 3–5 CKD compared to healthy subjects, by Papavasiliou et al. [28], with EPO treatment preventing the reduction in enzyme activity observed in patients not receiving EPO.

The results of these five studies concur to the fact that CKD progression leads gradually to a state of increased ROS production, which is reflected by the elevated levels in markers of lipid damage and the decrease in the concentration of antioxidant molecules and activity of antioxidant enzymes. As mentioned already, lipid peroxidation affects erythrocyte half-life, as its excess could overcome cell repair capacity [10]. According to Peuchant et al. [11], their results could be attributed to the fact that patients with CKD have decreased erythrocytes survival. Thus erythropoiesis is activated and could increase RBC precursors and enzymatic synthesis as well as consumption of antioxidant enzymes. Moreover, additional mechanisms are implicated in the enhanced lipid peroxidation in CKD such as the reduction in glucose-6-phosphate dehydrogenase activity, which leads to a decrease in NADPH and GSH concentrations and vitamin E deficiency [31–33].

Undeniably, a compromised antioxidant intake may contribute to the above observations, as predialysis patients need to adhere to specific diet guidelines and restrictions [27]. Indeed, intake of exogenous antioxidant vitamins, such as vitamins A and C, and glutathione precursors were found to be at lower levels in predialysis patients compared to healthy subjects, with nutritional intakes worsening as CKD became more severe and evident negative correlations between antioxidant intakes with oxidative stress levels [27].

### 4.2. Interventions in Predialysis Patients and Redox Status

Several interventions to correct for anemia, counteract inflammation, and supplement nutrition have become part of clinical and periclinical care in addition to appropriate pharmacological therapy and could affect redox balance. At the predialysis stage we located only four references that fulfilled our criteria.

Ganguli et al. [34] reported that various forms of intravenous iron [low molecular weight iron dextran (ID), sodium ferric gluconate complex, in sucrose (SFGC), and iron sucrose (IS)] at clinically used doses resulted in elevated MDA levels, measured immediately after the iron transfusion period [34]. Marsillach et al. [35] evaluated the 6-month effects of EPO treatment together with iron treatment (SFGC or IS), measuring antibodies against ox-LDL and paraoxonase (PON1) activity and concentration which is assumed to play an important role in oxidized lipid degradation. Results revealed decreased levels of ox-LDL antibodies and increased serum PON1 activity despite the iron treatment [35]. However, in addition to an effect of EPO, large differences in baseline LDL and ferritin levels between the two studied cohorts can explain the conflicting reports.

Ramos et al. [36] investigated whether the administration of mixed tocopherols and lipoic acid for 2 months could modify oxidative stress indices in CKD patients. However, no significant changes in F2-isoprostanes and protein thiols concentrations were observed in these patients compared to matching controls. As the authors discussed, the sample size, the intervention's duration, the dose, or the composition of antioxidants all could probably have been biologically ineffective in altering the redox status of this particular patient population [36]. Moreillon et al. [37] assessed the antioxidant effects of two types of herbal supplements curcumin and* Boswellia serrata*, on GPx levels, after 8-week supplementation. Despite the increase in plasma GPx levels in the treatment group, results were not statistically significant. Perhaps the duration of supplementation was not sufficient enough for the small sample size used [37] to detect an effect.

Data in non-CKD patients suggest that higher doses of vitamin E supplementation may be necessary to observe measurable changes in redox status. For example, in hypercholesterolemia patients, after 4 months of vitamin E supplementation, there was a decrease in F2-isoprostane concentration, only with the high doses of 1600 and 3200 IU/day [38]. Herbal supplements such as curcumin [39] and* Boswellia serrata* [40, 41] are known for their anti-inflammatory and antioxidant characteristics, for example, in patients with colitis [40, 41]. While one study [37] did not report an effect, future studies could explore further their possible contribution to ameliorate oxidative stress in predialysis patients via nutritional strategies.

Apart from the impaired nutritional status, CKD patients are also characterized by iron deficiency and depletion of iron storage. Although the administration of intravenous iron is fully recommended in these patients for anemia correction, the short-term safety and long-term safety of this administration remain unclear. According to Bishu and Agarwal [42] intravenous iron administration contributes to elevated oxidative stress and endothelial dysfunction in CKD patients. Moreover, according to Himmelfarb et al. [43], endothelial dysfunction plays a key role in the pathogenesis of atherosclerosis in CKD patients and oxidative stress is implicated in this pathway. It can be surmised that a treatment meant to correct anemia and iron supplementation could exacerbate atherosclerosis by promoting oxidative stress. The two studies supplementing iron to predialysis patients reviewed above, Ganguli et al. [34] and Marsillach et al. [35], reported conflicting effects of iron on lipid peroxidation. However, the interaction of iron to circulating LDL cannot be underestimated (i.e., a systemically atherogenic environment), while at the same time EPO appears to confer an antioxidant advantage (as shown earlier by Papavasiliou et al. [28] that EPO treatment prevented the reduction in erythrocytes GPx activity observed in patients not receiving EPO).

### 4.3. HD Patients and Redox Status

Overall, 14 studies were found, which examined the oxidant and antioxidant status in HD patients and compared them with healthy subjects (Table 2). These studies evaluated oxidative damage as it was assessed by lipid peroxidation markers MDA and isoprostanes and protein carbonylation as it was assessed by protein carbonyls and advanced oxidation protein products (AOPPs). Moreover, in these studies, the antioxidant defense was evaluated by measuring the antioxidant molecules sulfhydryl groups and GSH, the antioxidant enzymes SOD, GPx, and Gr reductase, and the total antioxidant capacity markers oxygen radical absorbing capacity (ORAC), trolox equivalent antioxidant capacity (TEAC), and total antioxidant capacity (TAC).

More specifically, in seven studies, increased levels of MDA [29, 44–49] were observed in HD patients compared to healthy individuals, reflecting extensive lipid damage. Sommerburg et al. [44] reported that HD patients who received long-term EPO treatment showed decreased levels of lipid peroxidation compared to patients who did not receive EPO treatment. Furthermore, Dolegowska et al. [50] reported a significant increase in plasma isoprostanes (8-iPF2a-III) concentrations in HD patients.

Regarding protein damage, levels of protein carbonyls were also found to be elevated in HD patients in four studies [49, 51–53]. In addition, advanced oxidation protein products (AOPPs) were found to be in high levels in HD patients in two studies [46, 53]; however, they were reported to be no different from control in one study [54]. In the latter study of Fragedaki et al. [54], patients were on dialysis for a shorter period (an average of 3 years) than the other studies [46, 53], a fact that could affect AOPPs levels. Finally, Choi et al. [30] reported increased XO activity in HD patients compared to healthy subjects (by almost 3 times), with XO activity being also higher (by 2.1 times) than predialysis patients. Taking into consideration the above results, there is evidence of extensive oxidative damage to total protein content and lipids in HD patients.

Regarding the antioxidant capacity in HD patients, the literature provides differing results. Sakata et al. reported a significant increase in “global” antioxidant capacity markers, such as ORAC and TEAC [46]. On the other hand, Dimitrijevic et al. [47] reported a significant decrease in total antioxidant capacity (TAC) of HD patients compared to controls, while decreased albumin levels were also observed. It should be noted that these global antioxidant capacity markers do not represent innate antioxidant capacity but rather represent global availability of free radical scavenging compounds, whose levels are greatly affected by nutrition.

Moreover, the activity of the free radical scavenging enzymes plasma GPx (GSH-Px) and SOD was found to be significantly decreased in HD patients [48]. GPx has been identified in two forms, cellular or cytosolic (GPx-1), which is presented in red blood cells and the cytosol of almost all tissues, and extracellular (GPx-3) in the plasma, with selenium (Se) being a basic structural component in both forms, [48], an element also affected by nutrition. Additionally, Gr reductase activity was found to be significantly higher in HD patients compared to healthy subjects [55], probably reflecting the organism's efforts to activate the antioxidant defenses. Likewise, that study also reported increased GSH total blood concentrations in the HD patients studied [55]. On the other hand, two studies [47, 53] reported a large reduction of free sulfhydryl groups' levels in HD patients, measured in plasma.

All the results concur to the fact that HD treatment seems to contribute to augmented oxidative stress. Most of the studies observed increased levels of protein and lipid damage in HD patients, which were measured with established biomarkers. As it appears, HD treatment causes an enhanced rate of LDL oxidation, which leads to the development of atherosclerosis, as reflected in elevated blood MDA levels. One mechanism is that the uremic milieu and the HD process* per se* stimulate neutrophils, a procedure known as “neutrophil burst,” which directly or indirectly results in hydrogen peroxide production. This is then converted to hypochlorous acid, which in turn contributes to the oxidation of plasma proteins and LDL, assaulting their membranes [46, 56]. Furthermore, as it was referred above, lipid peroxidation negatively affects erythrocyte half-life leading to decreased survival and manifestation of anemia. EPO appears to help as Sommerburg et al. [44] reported that HD patients who received long-term EPO treatment had decreased levels of lipid peroxidation. These results in HD concur with findings in predialysis patients, where also EPO appears to confer an antioxidant advantage [28], by preventing the reduction in erythrocytes GPx activity observed in patients not receiving EPO. Moreover, while erythropoiesis is, in a healthy individual, stimulated by ROS, as discussed by Migliaccio, erythropoiesis also counteracts circulating ROS levels via increases in available catalase and possibly other antioxidant enzymes [57]. Further, on the atherosclerosis mechanism, the presence of F2-isoprostanes has been determined in human atherosclerotic lesions [58]. F2-isoprostanes are created during the nonenzymatic peroxidation of arachidonic acid bound up with phospholipids of cells membranes and lipoproteins [59–61], and they are considered sensitive and specific indicators of oxidative stress intensity* in vivo* [58, 62]. Given reports, such as [50], of a significant increase in plasma isoprostanes in HD patients, the link between oxidative stress and CVD in HD patients is further highlighted.

The results on the antioxidant capacity of HD patients are not as straightforward to explain. Caloric restriction (without essential nutrient deficiency) in rats has been shown to result in decreased antioxidant capacity (ORAC) [63]. CKD patients, however, are subject to both caloric restriction and deficient antioxidant intakes [23]. Differences between studies reporting on HD antioxidant capacity could be thus attributed not only to adaptive mechanisms, due to years in dialysis and comorbidities, but also to dietary restrictions and interindividual differences in antioxidant nutritional intakes, as already reported in predialysis patients [27].

Additionally, the hemodialysis process* per se* may compromise key nutrient and trace mineral levels as highlighted with regard to levels of Se, a basic component of GPx. Guo et al. [48] reported that HD patients presented with lower plasma Se concentrations than healthy subjects and this could be attributed to either impaired diet/absorption or increased loss of Se during dialysis treatment [64]. Additionally, taking into account the fact that the kidney is the main site of plasma GPx synthesis and is also capable of Se accumulation [65], the fact that the levels of both Se and GPx are reduced in ESRD patients is not unexpected. A similar explanation could be also given for the significant reduction in plasma SOD activity observed in HD patients, linked to Zn availability, which is a structural element of SOD and whose levels tend also to be reduced in renal insufficiency [66].

Regarding GSH depletion in HD patients, many mechanisms have been proposed. According to Yawata and Jacob [31], an obstruction to the pentose phosphate pathway leading to impaired production of NADPH occurs in patients with ESRD. As a result, Gr reductase cannot recycle GSSG back to GSH using NADPH as electron source [31]. Notwithstanding, in many studies, GR activity has been found to be increased or to be in normal levels [12, 55, 67, 68]. Thus, GSH depletion could be alternatively attributed to a diminished GSH synthesis and/or to an increased GSH degradation, since its precursors cystine, glutamate, and glycine could be normal or could be elevated in patients' blood [69].

### 4.4. Redox Status before and after the HD Treatment

The hemodialysis treatment can affect patients' redox status, and our systematic literature search revealed 11 studies which examined the possible contribution of HD treatment to redox imbalance (Table 3). Three studies indicated that HD treatment* per se* augments lipid peroxidation, assessed by MDA [29, 70] and F2-isoprostanes levels [50], and induces protein damage, assessed by protein carbonylation [53]. However, there were studies which did not observe any significant changes in lipid peroxidation (unchanged MDA levels) [71, 72], F2-isoprostanes levels [73], and ox-LDL levels [74] following the HD process [71–74]. Moreover, in Malindretos et al.'s study, oxidized LDL levels did not significantly change after HD treatment, and intravenous iron administration during HD did not change its concentration following HD [74].

Regarding antioxidant capacity after completion of HD therapy, there are conflicting findings. Two studies reported that the levels of GSH and the activities of GPx and Gr reductase increased after HD therapy [55, 75]. However, there were three studies in younger patients which observed reduced levels of GSH [29, 70], reduced GPx activity [70], and reduced TAS levels [70, 76] following HD.

Ogunro et al. [70] evaluated the effects of cellulose and polysulfone membranes, which are the most commonly used types, on redox status in chronic HD patients. SOD activity was significantly reduced in both cellulose and polysulfone membrane dialysis. Regarding other antioxidant biomarkers evaluated in this study, both types of membranes caused changes to the same direction but not significantly in all cases. Specifically, CAT activity was significantly increased only in polysulfone membrane, while GPx activity was reduced in both types but not significantly. Additionally, TAS and GSH levels were significantly reduced after cellulose membrane HD, while the reductions were not significant in polysulfone membrane users. Lastly, following HD treatment, MDA levels were increased in both types but the change was significant only in cellulose membrane.

On the other hand, Ward et al. [53] did not find differing responses to the use of polysulfone or cellulose membranes on protein oxidation. These HD patients demonstrated already high plasma protein carbonyls and AOPP concentrations compared to normal values before HD treatment. With HD, using either membrane, protein carbonyls increased significantly over the course of HD and remained significantly elevated for the postdialysis period, indicating worsening in protein oxidative damage. Dialysis had no effect on AOPP levels and their values remained significantly higher than normal after dialysis. Perhaps counterintuitively, in that study, following HD a significant increase in plasma free sulfhydryl groups concentrations was found (levels essentially corrected to the levels of normal values) for both membranes used [53].

Bober et al. [29] assessed the effects of glucose content in the dialyzing fluid on RBC antioxidant capacity. They found that glucose concentrations of about 5.6 mmol/l resulted in augmentation of the hexose monophosphate cycle (HMP) in erythrocytes and accordingly benefited the antioxidant system. Furthermore, the group that received the glucose-free HD treatment presented with increased TBARS concentration after the treatment [29]. According to the authors, this probably occurred because the free radicals produced during the glucose-free HD session could not be neutralized through the nonenzymatic pathway at a satisfactory rate [29]. In the cross-sectional study of Dolegowska et al. [50], there were no significant differences either before or after HD treatment in 8-iPF2a-III levels in RBC membranes of HD patients treated with glucose or glucose-free dialyzing fluids. Still, they reported a significant increase of plasma 8-iPF2-III concentration following HD in the glucose-free group (which however started at a much lower level than the glucose treated group).

Various mechanisms have been proposed to account for the increased oxidative damage following HD treatment: the issue of hemoincompatibility induced by the contact between human blood and the nonbiological materials of the hemodialyzer and the resulting effects on leukocyte and platelet activation and inflammation [20, 77, 78], systemic inflammation effects mediated via neutrophil activity burst resulting in the release of ROS into the bloodstream [46, 56], and the role the HD process* per se* in reducing plasma antioxidant defense because several antioxidant dialyzable solutes are removed during HD.

The role of the type of dialysis membrane used is not straightforward [53, 71]. This is probably due to the different reactions that take place in sulfhydryl groups and protein carbonyl formation. Possibly, protein sulfhydryl groups are oxidized reversibly through small molecules oxidants, which can be removed by dialysis process, while AOPP and protein carbonyls may represent a chronic state of irreversible oxidative damage [53].

With regard to dialyzing fluid composition, it could be generally concluded that glucose has a beneficial effect on the antioxidant properties of RBC and protects them by decreasing the risk for hemolysis [29, 50].

### 4.5. Interventions in HD Patients and Redox Status

Three studies examined the effects of vitamin C administration on redox status of HD patients. Fumeron et al. [77] reported that oral administration of 250 mg vitamin C three times per week for two months could not either increase GSH or reduce protein carbonyls levels. Washio et al. [78] found that oral administration of vitamin C for 3 months ranging from low (200 mg) to high dose (1000 mg) could not suppress the enhancement of Cu/Zn-SOD expression, an oxidative stress marker. On the other hand, Tarng et al. [79] found that intravenous administration of 300 mg vitamin C for 8 weeks decreased significantly 8-OHdG contents in cellular DNA of lymphocytes. Moreover, in the same study, vitamin C significantly reduced intracellular ROS production of lymphocytes of patients on HD [79].

Regarding vitamin E, another important antioxidant substance, Smith et al. [73] examined the effects of 400 IU administration for 2 months on redox status of 11 HD patients. The elevated free F2-isoprostane plasma concentrations were not decreased by vitamin E supplementation. Likewise, treatment with a combination of mixed tocopherols plus *α*-lipoic acid (oral) for 6 months did not change plasma F2-isoprostane and F2-isofuran of HD patients compared to placebo group as reported by Himmelfarb et al. [80].

A pilot study by Trimarchi et al. [71] showed that the daily administration of 1,200 mg NAC for one month could significantly reduce plasma lipid peroxidation of HD patients compared to the control group. Finally, Fatouros et al. [81] reported that daily administration of 20 mg/kg L-carnitine in 12 HD patients for 8 weeks resulted in 2,7-fold increased GSH/GSSG ratio, a 4.5% increase in glutathione peroxidase activity, a 19% decrease in MDA levels, and 27% decrease in protein carbonyls concentration.

Vitamin C is one of the most important water-soluble antioxidants. There is evidence that HD patients exhibit a 30–50% decrease in vitamin C levels after dialysis treatment, as reviewed elsewhere [82]. Moreover, reduced vitamin C levels in HD patients have been associated with an increased risk for CVD morbidity and mortality [83]. However, elsewhere, only intravenous administration was found to have a measurable beneficial effect [79]. Thus, oral administration of vitamin C may not be sufficient to correct its deficiency in HD.

Oral administration of vitamin E did not decrease plasma isoprostanes in HD patients [74, 83]. In contrast, it has been reported that long-term administration of 200 mg of vitamin E decreased F2-isoprostanes plasma levels in mildly hypercholesterolemic men [84]. Moreover, in healthy subjects, vitamin E administration resulted in lower F2-isoprostane concentrations in urinary samples [85]. Perhaps differences in outcomes relate to dosage and duration of administration could explain these results, notwithstanding the augmented oxidant production characterizing HD patients in which perhaps oral administration of vitamin E, at safe levels for these patients, could not combat.

N-acetylcysteine (NAC) is a ROS scavenger and its administration seems to increase glutathione concentration given that it raises intracellular levels of one of its precursors, cysteine [86–88]. Along with L-carnitine, these products showed promising results and should be further examined in larger patient cohorts.

### 4.6. Other Types of Dialysis and Redox Status

Our systematic literature review revealed 4 studies that examined other types of dialysis in relation to oxidative stress and antioxidant status biomarkers (Table 4). A metabonomics study by Choi et al. [30] examined the effects of different dialysis modalities on serum profiles of patients. XO activity levels were similar for both HD and peritoneal dialysis (PD) patients. Still XO activity was significantly higher in PD patients compared to controls and to nondialyzed uremic patients (non-HD or PD) [30], perhaps justifying the need to address it pharmaceutically (see below in* Interventions in PD*).

In the study of Canestrari et al. [89] erythrocyte GSH levels were unchanged or slightly increased in the patients compared to the control group, while a nearly 3-fold increase in GSSG was observed in the PD group [89]. In the same study, plasma GSH levels were significantly decreased in the PD group compared to control group and GSSG levels were slightly increased in PD patients [89]. Moreover, increased RBC and plasma TBARS levels were found in PD patients compared to control group. Finally, significantly higher erythrocyte GSH-Px activity was observed in the PD group, while plasma GSH-Px activity was similar in PD patients and controls [89].

Moreover, a study by González-Diez et al. [90] examined and compared the effects of haemodiafiltration (HFR) on HD with polysulfone membranes (HD-PS). The HFR group demonstrated moderate changes in oxidative stress biomarkers and antioxidant capacity markers, indicating that this method may in the long term preserve a more stable and balanced redox status compared to HD-PS [90].

### 4.7. Interventions in PD Patients and Redox Status

Imani et al. [91] evaluated the effects of soy consumption (28 g/day) for 8 weeks on oxidative stress in PD patients. Results indicated no changes in serum ox-LDL between soy and control groups of PD patients; however, an improvement in thrombosis risk was reported. The fact that 78% of the specific peritoneal dialysis patients received vitamin C and/or E could explain the lack of a measurable effect. We do consider, however, that such dietary interventions as well as antioxidant interventions of adjunct pharmaceuticals need further examination (see below).

Overall, based on the limited data reviewed so far, PD also appears to be associated with increased oxidative stress, with Choi et al.'s study [30] showing PD patients to have as high XO activity as HD patients and Canestrari et al.'s study [89] showing not only increased oxidative stress but also evidence of reductions in antioxidant capacity, compared to controls. As XO is a superoxide producing enzyme its inhibition has been viewed as a way to reduce CVD risk (e.g., via allopurinol, [92]). To circumvent possible allopurinol's toxicity in renal insufficiency patients, careful screening and tolerance protocols are needed [93]. A new nonpurine alternative, febuxostat, has been shown to reduce serum levels of uric acid and levels of a marker of DNA oxidation (8-oxo-dG) following 6-month administration in HD patients [94].

In non-CKD patients, soy consumption has been reported to improve redox status (e.g., in gestational diabetes [95]). Notably, the combination of soy milk with* Lactobacillus plantarum* was recently shown not only to improve redox status, versus consumption of plain soy milk, but also to exert a promising epigenetic effect on the DNA repair capability of type II diabetic patients [96]. Dietary antioxidants may play a role even before disease diagnosis, whether independently or as a component of a generally “healthy lifestyle,” as increased albuminuria (a prognostic marker for kidney disease) has been found to be associated with reduced levels of diet-derived carotenoids in an indigenous Australia population [97]. Such and other findings highlight the complexity and overarching effect on redox status introduced by diet. We suspect that, whether early in the disease or towards advanced stages (especially when CKD patients face severe nutritional restrictions when at the same time not only noxious but also potentially beneficial antioxidant intermediates may accumulate [98]), the role of targeted nutritional aids to reduce systemic oxidative stress could prove to be crucial for the reduction of CVD and malignancies risk.

Our evaluation of the systemic effects of oxidative stress in CKD patients does not account for the issue of kidney tissue and cell specific disturbances in redox homeostasis. Although beyond the aim of the present systematic review, we consider it of interest to give some highlights on the role of kidney injury* per se*. Initial glomerular injury affects podocytes, the glomerular visceral epithelial cells, which constitute not only the target but also the source of ROS. Bek et al. 2003 [99], using cultured mouse podocytes, demonstrated that STRA13, a prostaglandin E_2_-induced gene, protected the redox homeostasis in this cell type, preventing podocytes from injuries and subsequent detrimental effects on kidney functionality. Apart from podocytes, another type of kidney cells, known as mesangial cells, is known to play an important role in CKD progression. More specifically, angiotensin II (ANG II) stimulates proliferation of the mesangial cells resulting in pathological changes in CKD [100, 101]. Although the exact mechanism remains incompletely understood, Ding et al. 2007 [102] reported that the ROS-epidermal growth factor receptor EGFR-JNK pathway is involved in transducing the proliferative effects of ANG II in cultured mesangial cells. Thus, specifically targeting the injured kidney to ameliorate or break the vicious circle of recurrent injury, inflammation, and oxidative stress and salvage glomerular function (as suggested in [103]) holds promise.

## 5. Conclusions

The results of this systematic review indicate that oxidative stress is implicated in the CKD pathophysiology and as the kidney function is getting worse, the redox status imbalance becomes more profound. Even at an early disease stage, lipid peroxidation markers are elevated. This premature development sets perhaps the pace for accelerated atherogenesis, which appears to explain the profoundly negative CVD risk of end stage patients. Thus early interventions to combat lipid peroxidation in predialysis patients need to be considered.

At the end stage, patients present with both overabundance of ROS and mostly compromised antioxidant capacity. HD therapy* per se* seems to contribute to the oxidant and antioxidant imbalance. Iron supplementation has a prooxidant role but appears largely balanced out by appropriate EPO supplementation. The choice of dialysis membrane and dialyzing fluid glucose content may modulate the prooxidant effect of HD treatment. Moreover, antioxidant supplements may ameliorate oxidative stress levels by enhancing antioxidants defense; however, mode of administration (i.e., intravenous for vitamin C) should be carefully considered for efficacy. Vitamin E supplementation has been so far shown to be ineffective in HD patients; however, small studies testing NAC and L-carnitine reported promising results.

Peritoneal dialysis did not appear to have a clear advantage over HD treatment, despite the biocompatibility advantage, while HFR may better preserve some antioxidant molecules. However, the small number of studies in PD and HFR does not allow for safe conclusions and further work is needed to clarify the role of dialysis modality on redox status of ESRD patients.

Future studies addressing possible interventions to reduce the high burden of oxidative stress in patients with CKD are needed. Such studies should be prospective, comparative, and systematically structured according to outcome, should include a large number of patients, and should have an adequately long follow-up.

## Figures and Tables

**Table 1:** Redox status in predialysis CKD patients.

Authors	Groups	MDA	XO	GSH	SOD	CAT	GPx
Peuchant et al., 1996 [11]	(a) 16 CKD patients (age: 43.6 ± 11.2 yr) GFR: 12.6 ± 5.4 mL/min (b) 26 healthy subjects (age: 43.6 ± 11.2 yr)	RBC-free MDA: (a) ↑ 3.88 ± 2.67 (b) 1.74 ± 0.56 Total MDA: (a) 10.0 ± 9.31 (b) 7.85 ± 2.82 (nmol/mL)			(a) 800 (b) 790 (U/g Hb)	(a) 45 (b) 43 (U/g Hb)	(a) 34 (b) 32 (U/g Hb)

Papavasiliou et al., 2005 [28]	(a) 12 CKD patients (age: 66 yr) Creatinine clearance: 23.9 ± 6.6 mL/min (b) 11 CKD patients (age: 61 yr) Creatinine clearance: 20.2 ± 6.5 mL/min (c) 13 CKD patients (age: 63 yr) Creatinine clearance: 87.2 ± 7.1 mL/min (d) 15 healthy subjects (age: 59 yr)	Plasma: (a) ↑ 1.2 (b) ↑ 1.25 (c) 1.15^††^ (d) 0.9 (nmol/mL)					(a) ↑ 15.50 ± 5 (b)↑ 15.20 ± 4.70 (c) ↑ 11.30 ± 2.88 (d) 9.98 ± 3.33 (IU/g Hb)

Bober et al., 2010 [29]	(a) 21 CKD patients (age: 56.8 ± 16.0 yr) (b) 21 healthy subjects (age: 56 ± 16.6 yr)	Plasma: (a) 1.05 ± 0.21 (b) 0.79 ± 0.12 (*μ*mol/L)		(a) ↑ 8.26 ± 1.16 (b) 7.26 ± 1.50 (nmol/g Hb)			

Choi et al., 2011 [30]	(a) 16 CKD patients GFR: 23.56 ± 10.64 mL/min/1.73 m^2^ (b) 18 healthy subjects (age: 48.9 ± 15.4 yr)		(a) ↑ 6.3 ± 1.5 (b) 4.5 ± 0.9 (RLU)				

Sahni et al., 2012 [27]	(a) 60 CKD patients (age: 43.05 ± 11.9 yr) GFR: 12.75 ± 5.52 mL/min (b) 85 CKD patients (age: 42.88 ± 11.4 yr) GFR: 28.61 ± 12.93 mL/min (c) 40 Healthy subjects (age: 42.42 ± 2.2 yr)	RBC: (a) ↑ 4.89 ± 1.33^†^ (b) ↑ 2.77 ± 1.015 (c) 1.13 ± 0.219 (*μ*mol/g Hb)		(a) ↓ 0.61 ± 0.40^†^ (b) ↓ 1.05 ± 0.334 (c) 1.87 ± 0.608 (*μ*mol/g Hb)	(a) ↓ 994.57 ± 87.14^†^ (b) ↓ 1136.99 ± 101.56 (c) 1382.55 ± 93.93 (IU)	(a) ↓ 125.86 ± 17.36^†^ (b) ↓ 139.19 ± 24.35 (c) 184.66 ± 17.29 (*μ*L/min/mL Hb)	

MDA: malondialdehyde; XO: xanthine oxidase; GSH: reduced glutathione; SOD: superoxide dismutase; CAT: catalase; GPX: glutathione peroxidase; yr: years; ↑↓: versus healthy controls; ^†^difference between a and b groups; ^††^difference between a and b groups with c group; ± stands for standard deviation.

**Table 2:** Redox status in HD patients.

Authors	Groups	HD modality and period	TBARS/MDA	Free sulfhydryl groups	Isoprostanes 8-iPF2a-III	AOPP	Protein carbonyls	XO	GSH	ORAC/TEAC/TAC	SOD/GPx/GR reductase
Haklar et al., 1995 [49]	(a) 14 HD patients (age: 52 yr) (b) 14 healthy subjects (age: 40 yr)	Cuprophan, dialyzers, 22 months, 3x/week/4 h	MDA (nmol/mL) (a) **↑**5.50 ± 0.6 (b) 2.82 ± 0.36				(a) ↑41.2 ± 10.4 (b) 22.6 ± 5.5 (nmol/mg protein)				

Sommerburg et al., 1998 [44]	(a) 8 HD patients, Hb < 10 g/dL (age: 58 yr) (b) 8 HD patients, Hb > 10 g/dL (age: 62 yr) (c) 27 HD patients receiving rHuEpo (age: 66 yr) (d) 20 healthy subjects (age: 59 yr)	Bicarbonate HD, 6–10 years, 3x/week/4-5 h	MDA (*μ*M) (a) **↑**3.81 ± 0.86 (b) **↑**2.77 ± 0.58 (c) **↑**2.50 ± 0.12 (d) 0.37 ± 0.03								

Ward et al., 2003 [53]	(a) 11 HD patients (age: 51 ± 5 yr) (b) 12–17 healthy subjects (age 23–54 yr)	49 ± 11 months		(a) ↓ 268 ± 22 (b) 438 ± 16 (*μ*mol/L)		(a) ↑191 ± 27 (b) 74 ± 8 (*μ*mol/L)	(a) ↑0.144 ± 0.037 (b) 0.041 ± 0.008 (nmol/mg)				

Choi et al. [30]	(a) 18 patients (age: 52.0 ± 17 yr) (b) 16 healthy subjects (age: 48.9 ± 15.4 yr)	94.3 ± 43.5 months, 3x/week/4 h						(a) ↑13 ± 9.4 (b) 4.5 ± 0.9 (RLU)			

Triolo et al., 2003 [45]	(a) 10 HD patients (age: 64.6 ± 13.7 yr) (b) 30 healthy subjects (age: 59.8 ± 9.4 yr)	Bicarbonate HD, 86.6 ± 47.2 months, 3x/week/3.5–4 h	MDA (nmol/mL) (a) **↑**1.87 ± 0.36 (b) 1.13 ± 0.18								

Anraku et al., 2004 [52]	(a) 11 HD patients (age: 25 to 87 yr) (b) 11 age-matched healthy subjects	Bicarbonate, 1–9 years, 3x/week/4-5 h					(a) **↑**1.0 ± 0.1 (b) 0.4 ± 0.03 (nmol/mg protein)				

Fragedaki et al., 2005 [54]	(a) 12 SHD patients (age: 57.5 ± 12.8 yr) (b) 13 MHD patients (age: 48 ± 11.5 yr) (c) 12 healthy subjects (age: 52.9 ± 10.7 yr)	(a) Polyethersulfone, 3.6 ± 1.82 years, 3x/week/4–4.5 h (b) Polyethersulfone, 3.3 ± 1.37 years, 6x/week/2–2.5 h				(a) 0.44 ± 0.23 (b) 0.57 ± 0.36 (c) 0.60 ± 0.46 (nmol/mg protein)					

Mera et al., 2005 [51]	(a) 20 HD patients (age: 62.8 ± 12.7 yr) (b) 10 healthy subjects (age: 67.8 ± 1.8 yr)	Bicarbonate HD, 1–9 years, 3x/week/4-5 h					(a) ↑3.12 ± 1.1 (b) 2.10 ± 0.34 (nmol/mg protein)				

Stepniewska et al., 2006 [55]	(a) 25 HD patients (age: 50.3 ± 13.79 yr) (b) 26 HD patients (age: 60.54 ± 13.54 yr) (c) 29 healthy subjects (age: 49.7 ± 11.4 yr)	(a) Polysulfone HD, glucose-free fluid, 27.44 ± 15.87 months, 3x/week, 4 h (b) Polysulfone HD, glucose fluid, 24.43 ± 12.68 months 3x/week/4 h							(a) **↑**19.6 ± 8.8 (b) ↑20.5 ± 8.0 (c) 14.2 ± 2.1 (*μ*mol/g Hb)		Gr reductase (a) **↑**2.82 ± 0.98 (b) **↑**2.57 ± 0.76 (c) 1.95 ± 0.40 (U/g Hb)

Dolegowska et al., 2007 [50]	(a) 22 HD patients (age: 53.06 ± 11.43 yr) (b) 22 HD patients (age: 57.70 ± 14.78 yr) (c) 22 healthy subjects (age: 51.93 ± 9.94 yr)	(a) Polysulfone HD, glucose-free fluid, 3x/week/4 h (b) Polysulfone HD, glucose fluid, 3x/week/4 h			(a) Plasma: 0.05 ± 0.02 RBC: 7.19 ± 10.45 (b) ↑Plasma: 0.19 ± 0.15 RBC: 8.75 ± 7.58 (c) Plasma: 0.10 ± 0.05 RBC: 5.29 ± 7.54 (ng/mL), (ng/g Hb)						

Sakata et al., 2008 [46]	(a) 36 HD patients (age: 63.6 ± 12.1 yr) (b) 15 healthy subjects (age: 32.5 ± 8.6 yr)	Bicarbonate HD, 12.1 ± 7.0 years	MDA (*μ*M) (a) **↑**0.25 ± 0.11 (b) 0.05 ± 0.02			(a) ↑88.8 ± 39.7 (b) 43.8 ± 20.3 (*μ*M)				(a) ORAC: ↑2,672 ± 554 TEAC: ↑0.77 ± 0.2 (b) ORAC: 1,363 ± 174 TEAC: 0.43 ± 0.12 (U/mL), mM Trol eq/L	

Bober et al., 2010 [29]	(a) 22 HD patients (age: 55.9 ± 14.8 yr) (b) 23 HD patients (age: 64.3 ± 12.1 yr) (c) 21 healthy subjects (age: 56 ± 16.6 yr)	(a) Polysulfone HD, glucose-free fluid, 8.78 ± 6.42 months, 3x/week (b) Polysulfone HD, glucose fluid, 9.45 ± 6.62 months 3x/week	MDA (a) **↑**1.56 ± 0.27 (b) **↑**1.36 ± 0.30 (c) 0.79 ± 0.12 (*μ*mol/L)								

Dimitrijevic et al., 2012 [47]	(a) 15 HD patients (age: 55.6 ± 18.2 yr) (b) 29 healthy subjects (age: 55 ± 15.8 yr)	Bicarbonate HD, 52.5 ± 61.6 months	MDA (a) Plasma: **↑**11.3 ± 11.2 RBC: **↑**14.7 ± 2.2 (b) Plasma: 6.0 ± 1.1 RBC: 8.7 ± 1.3 (mmol/L), (nmol/mL)	(a) **↓**284.4 ± 44.5 (b) 449.2 ± 66.6 (*μ*mol/L)						(a) TAC: ↓2.5 ± 0.3 (b) TAC: 3.6 ± 0.5 (*μ*mol/L)	

Guo et al., 2013 [48]	(a) 20 HD patients (age: 55 ± 7 yr) (b) 25 healthy subjects (age: 53 ± 7 yr)	HD, 6 ± 1 years, 3x/week/4 h	MDA (a) **↑**6 (median) (b) 2.9 (nmol/L)								(a) GPx: ↓50.5 ± 8.4 SOD: ↓ 3.4 (b) GPx: 85.2 ± 6.1 SOD: 8.3 (U/mL) (median)

TBARS: thiobarbituric acid reactive substances; MDA: malondialdehyde; AOPP: advanced oxidation protein products; GSH: reduced glutathione; ORAC: oxygen radical absorbance capacity; TEAC: trolox equivalent antioxidant capacity; TAC: total antioxidant capacity; SOD: superoxide dismutase; GPX: glutathione peroxidase; yr: years; ↑↓: versus healthy controls; ± stands for standard deviation except in cases of studies of Ward et al. (2003) [53] and Anraku et al. (2004) [52] where ± stands for standard error.

**Table 3:** Redox status before and after the HD treatment.

Authors	Groups	HD modality and period	AOPP	Ox-LDL	Free sulfhydryl	Protein carbonyls	TBARS/MDA	F2-isoprostanes	GSH	SOD	GPx	GR reductase	CAT	TAS
Ward et al., 2003 [53]	11 HD patients, (age 51 ± 5 yr)	(a) polysulfone membrane (b) cellulose membrane 49 ± 11 months	(a) 190 ± 37 (b) 163 ± 26 (*μ*mol/L)		(a) ↑425 ± 15 (b) ↑408 ± 23 (*μ*mol/L)	(a) ↑0.175 ± 0.03 (b) ↑0.178 ± 0.04 (nmol/mg)								

Westhuyzen et al., 2003 [75]	13 HD patients, (age: 69.2 ± 15.6 yr)	Vitamin E membrane, 3x/wk (a) Baseline (b) At 6 wk (c) At 13 wk							(a) 0.99 ± 0.17 (b) 1.08 ± 0.16 (c) 1.04 ± 0.20 (mmol/L)	(a) 725 ± 102 (b) 746 ± 130 (c) 771 ± 151 (U/g Hb)	(a) 46.8 ± 14.8 (b) ↑55.7 ± 14.8 (c) ↑56.7 ± 17.2 (U/g Hb)			

Trimarchi et al., 2003 [71]	12 HD patients, (age: 65.5 ± 13.1 yr)	Cellulose membrane, for 20.83 months, 3x/wk					4.62 ± 0.9 (*μ*mol/L)							

Smith et al., 2003 [73]	11 HD patients, (age: 64 ± 4 yr)	Polysulfone membrane, 3x/wk						602 ± 105 (pg/mL)						

Stepniewska et al., 2006 [55]	(a) 25 HD patients, (age: 50.3 ± 13.7 yr) (b) 26 HD patients (age: 60.5 ± 13.5 yr)	(a) Polysulfone membrane, glucose-free fluid, for 27.44 ± 15.87 months, 3x/week/4 h (b) Polysulfone glucose fluid, for 24.43 ± 12.68 months, 3x/week/4 h							(a) ↑18.3 ± 7.2 (b) 19.8 ± 8.2 (mmol/g Hb)			(a) 2.69 ± 0.95 (b) ↑2.05 ± 0.59 (U/g H)		

Huang et al., 2006 [76]	25 HD patients, (age: 58 ± 3 yr)	HD for 12 months												↓0.5 (mmol/L)

Malindretos et al., 2007 [74]	20 HD patients, (age: 64.7 ± 17.3 yr):	Polysulfone membrane, 3x/wk/4 h		14.52 ± 8.3 (mU/L)										

Dolegowska et al., 2007 [50]	(a) 22 HD patients, (age: 53.1 ± 11.4 yr) (b) 22 HD patients, (age: 57.7 ± 14.7 yr)	(a) Polysulfone membrane, glucose-free fluid, 3x/week/4 h (b) Polysulfone glucose fluid, 3x/week/4 h						(a) ↑Plasma: 0.18 ± 0.17 RBC: 7.40 ± 11.22 (b) Plasma: 0.16 ± 0.10 RBC: 7.62 ± 7.03 (ng/mL), (ng/g Hb)						

Ramos and Martínez-Castelao, 2008 [72]	34 HD patients, (age: 57 ± 1 yr)	Cellulose membrane					0.28 ± 0.19 (ng/g HDL)							

Bober et al., 2010 [29]	(a) 22 HD patients, (age: 55.9 ± 14.8 yr) (b) 23 HD patients, (age: 64.3 ± 12.1 yr)	(a) Polysulfone membrane, glucose-free fluid, 3x/week (b) Polysulfone HD glucose fluid, 3x/week					(a) ↑2.01 ± 0.55 (b) ↓1.17 ± 0.31 (*μ*mol/L)		(a) ↓8.82 ± 2.22 (b) ↑11.07 ± 3.28 (nmol/g Hb)					

Ogunro et al., 2014 [70]	(a) 35 HD patients, (age: 51.9 ± 12.4 yr) (b) 38 HD patients, (age: 49.8 ± 10.6 yr)	(a) Cellulose membrane (b) Polysulfonate membrane, 3x/wk/4-5 h					(a) ↑6.05 ± 0.9 (b) 4.71 ± 0.7 (nmol/mL)		(a) ↓2.09 ± 0.3 (b) 3.68 ± 0.2 (*μ*mol/g Hb)	(a) ↓378 ± 13.2 (b) ↓418 ± 19.7 (U/g Hb)	(a) 22.5 ± 3.1 (b) 27.5 ± 4.2 (U/g Hb)		(a) 1231 ± 41.6 (b) ↑1370 ± 39.4 (U/g Hb)	(a) ↓1.0. ± 0.7 (b) 1.16 ± 0.2 (mmol/L trolox)

AOPP: advanced oxidation protein product; Ox-LDL: oxidized low density lipoprotein; MDA: malondialdehyde; GSH: reduced glutathione; SOD: superoxide dismutase; GPX: glutathione peroxidase; CAT: catalase; TAS: total antioxidant status; yr: years; ↑: versus before the HD treatment; ↓: versus before the HD treatment; ± stands for standard deviation except in cases of studies of Ward et al. (2003) [53] and Huang et al. (2006) [76] and Smith et al. (2003) [73] where ± stands for standard error.

**Table 4:** Other modalities of HD and redox status.

Authors	Groups	HD modality	TBARS/MDA	Protein carbonyls	GSSG	GSH	SOD	GPx	XO	CAT	TAC
Canestrari et al., 1995 [89]	(a) 18 patients (age: 62.9 ± 13.9 yr) (b) 15 healthy subjects (age: 59.2 ± 8.3 yr)	Peritoneal dialysis for 26.56 ± 23.27 months	(a) ↑RBC: 10.8 ± 3.8 Plasma: ↑74.5 ± 20.5 (b) RBC: 4.8 ± 2.6 Plasma: 46.6 ± 16.1 (nmol/g Hb or protein)		(a) RBC: ↑0.039 ± 0.014 Plasma: 0.011 ± 0.006 (b) RBC: 0.012 ± 0.011 Plasma: 0.009 ± 0.005 (*μ*mol/g Hb or protein)	(a) RBC: 6.40 ± 1.36 Plasma: ↓1.53 ± 0.69 (b) RBC: 4.97 ± 0.78 Plasma: 2.07 ± 0.20 (*μ*mol/g Hb or protein)		(a) RBC: ↑48.7 ± 11.5 Plasma: 5.02 ± 1.36 (b) RBC: 36.1 ± 8.5 Plasma: 5.50 ± 0.79 (U/g Hb or protein)			

Choi et al., 2011 [30]	(a) 18 patients (age: 48.1 ± 16.4 yr) (b) 18 patients (age: 52.0 ± 17 yr) (c) 16 healthy subjects (age: 48.9 ± 15.4 yr)	(a) Peritoneal dialysis (PD) for 99.8 ± 39.4 months, 1.7x/wk (b) Typical HD for 94.3 ± 43.5 months, 3x/wk							(a) ↑12.2 ± 3.5 (b) 13.0 ± 9.4 (c) 4.5 ± 0.9 (RLU)		

González-Diez et al., 2012 [90]	(a) 25 patients (b) 15 patients (age: —)	(a) Haemodialfiltration (HFR) for 1 year (b) HD with polysulfone membrane for 1 year	(a) ~19 (b) ~17 (*μ*M)	(a) ~2.2 (b) ~2.2 (nmol/mg protein)		(a) ~1 (b) ~0.9 (*μ*moles/g)	(a) ~2100 (b) ↓~1400 (U/g protein)	(a) ~42 (b) ~34 (U/g protein)		(a) ~18 (b) ~25 (U/g protein)	(a) ~4 (b) ~4 (mM Trolox)

TBARS: thiobarbituric acid reactive substances; Ox-LDL: oxidized low density lipoprotein; GSSG: oxidized glutathione; GSH: reduced glutathione; SOD: superoxide dismutase; GPx: glutathione peroxidase; XO: xanthine oxidase; CAT: catalase; TAC: total antioxidant capacity; yr: years; ↑: versus healthy, ↓: versus healthy, ~: values were estimated according to figures; ± stands for standard deviation.
